# Sustainable production of multimeric and functional recombinant human adiponectin using genome-edited chickens

**DOI:** 10.1186/s13036-024-00427-2

**Published:** 2024-05-07

**Authors:** Eunhui Yoo, Hee Jung Choi, Jin-Kyoo Kim, Young Min Kim, Jin Se Park, Jae Yong Han

**Affiliations:** 1 Department of Agricultural Biotechnology and Research Institute of Agriculture and Life Sciences, College of Agriculture and Life Sciences, Seoul National University, 1 Gwanak-ro, Gwanak-gu, Seoul, 08826 Republic of Korea; 2 Department of International Agricultural Technology & Institute of Green BioScience and Technology, Seoul National University, Pyeongchang, 25354 Gangwon-do Republic of Korea; 3 Avinnogen Co., Ltd, Seoul, Republic of Korea

**Keywords:** Adiponectin, Chicken bioreactor, Chicken oviduct magnum, Genome-edited chicken, High molecular weight ADPN, Multimeric ADPN

## Abstract

**Background:**

Adiponectin (ADPN) plays a critical role in endocrine and cardiovascular functions, but traditional production methods, such as *Escherichia coli* and mammalian systems, have faced challenges in generating sufficiently active middle molecular weight (MMW) and high molecular weight (HMW) forms of recombinant human ADPN (hADPN). In our previous study, we proposed genome-edited chickens as an efficient platform for producing multimeric hADPN. However, the consistency of multimeric hADPN expression in this system across generations had not been further investigated.

**Results:**

In this study, subsequent generations of ovalbumin (*OVA*) *ADPN* knock-in chickens showed stable multimeric hADPN production, yielding ~ 26% HMW ADPN (0.59 mg/mL) per hen. Comparative analysis revealed that egg white (EW)-derived hADPN predominantly consisted of hexameric and HMW forms, similar to serum-derived hADPN. In contrast, hADPN obtained from human embryonic kidney (HEK) 293 and High-Five (Hi-5) cells also exhibited the presence of trimers, indicating variability across different production systems. Furthermore, transcriptional expression analysis of ADPN multimerization-associated endoplasmic reticulum chaperone genes (*Ero1-Lα*, *DsbA-L*, *ERP44*, and *PDI)* indicated upregulation in the oviduct magnum of *ADPN* KI hens, suggesting the chicken oviduct magnum as the optimal site for HMW ADPN production. Lastly, the functional analysis demonstrated that EW-derived hADPN significantly reduced lipid droplets and downregulated lipid accumulation-related genes (*LOX-1*, *AT1R*, *FAS*, and *FABP4*) in human umbilical vein endothelial cells (HUVECs).

**Conclusion:**

In summary, stable and functional multimeric hADPN can be produced in genome-edited chickens even after generations. This highlights the potential of using chicken bioreactor for producing various high-value proteins.

**Supplementary Information:**

The online version contains supplementary material available at 10.1186/s13036-024-00427-2.

## Introduction

Adiponectin (ADPN) is a versatile hormone secreted by adipocytes, with diverse roles in endocrine and cardiovascular functions [1]. It exists in various multimeric forms, with the high molecular weight (HMW) form considered the most biologically active in mediating the insulin-sensitizing effects [2]. ADPN exerts protective effects against atherosclerosis and lipid accumulation by inhibiting the uptake of low-density lipoprotein (LDL) in endothelial cells [3–5]. Moreover, the ratio of HMW ADPN level to total ADPN level is closely correlated with the risk of atherosclerosis and endothelial dysfunction [6–8]. The oxidized low-density lipoprotein receptor 1 (*LOX-1*) plays a significant role in internalizing oxidized LDL, with its expression being upregulated upon the uptake of oxidized LDL [9]. It has been reported that oxidized LDL upregulates angiotensin II type 1 receptor (*AT1R*) mRNA via NADPH oxidase-mitogen-activated protein kinase-nuclear factor-kappaB pathway, along with *LOX-1* [10]. Additionally, fatty acid synthase (*FAS*) and fatty acid-binding protein 4 (*FABP4*), involved in lipogenesis and intracellular lipid accumulation, are downregulated in response to decreased lipid accumulation. Conversely, during hyperlipidemia, their expression levels are upregulated, indicating their involvement in lipid metabolism [11, 12].

The process of ADPN multimerization and secretion involves intricate regulation by molecular chaperones within the endoplasmic reticulum (ER), including disulfide bond A oxidoreductase-like protein (*DsbA-L*), ER oxidoreductase 1 alpha (*Ero1-Lα*), ER protein 44 (*ERP44*), and protein disulfide isomerase (*PDI*) [13]. *Ero1-Lα* facilitates HMW ADPN secretion, with *DsbA-L* and *ERP44* are involved in ADPN multimerization [14]. *PDI* is essential for intermolecular disulfide bond formation and adiponectin multimerization [15].

The clinical importance of HMW ADPN over low molecular weight (LMW) ADPN for optimal functionality is well-established [7, 16, 17]. However, challenges persist in the production of recombinant human ADPN (hADPN), particularly in achieving stable multimeric isoforms, limiting its therapeutic potential. Conventional production methods using *Escherichia coli* (*E. coli*) or mammalian cell-based systems often yield predominantly LMW forms due to insufficient post-translational modifications [18]. Bacterially produced ADPN, primarily in monomeric form, lacks biological activity and cannot effectively regulate glucose metabolism [19]. Therefore, in vivo studies often rely on mouse serum-derived ADPN, enriched in HMW multimers, for functional assessments [20–22].

Chickens represent a promising model for ADPN production due to their naturally occurring hyperglycemia and insulin resistance, resulting in elevated serum levels of HMW ADPN [23–25]. Furthermore, hyperglycemia counteracts the suppressive effect of hyperinsulinemia on plasma ADPN levels in humans, providing chickens with a unique advantage in ADPN levels due to their naturally occurring hyperglycemic and insulin-resistant state [26–28]. Recently, functional hADPN, predominantly in multimeric forms, was successfully produced using chickens through ovalbumin (*OVA*)-targeted clustered regularly interspaced short palindromic repeats (CRISPR)/CRISPR-associated protein 9 (Cas9) gene editing [29].

Therefore, in this study, we aim to evaluate the sustainability of multimeric hADPN production across subsequent generations of genome-edited chickens. Additionally, we compared the multimeric characteristics of hADPN produced in chickens with those generated by other recombinant systems, supplemented by a comprehensive transcriptional analysis of genes associated with multimerization. Finally, we explored the functional effects of different recombinant hADPN on inhibiting lipid accumulation in human umbilical vein endothelial cells (HUVECs).

## Materials and methods

### Experimental animals and animal care

The care and experimental use of chickens were approved by the Institute of Laboratory Animal Resources, Seoul National University, and conducted in accordance with Animal Research: Reporting of In Vivo Experiments (ARRIVE) guidelines. Chickens were maintained following standard procedures at the University Animal Farm. Methods were approved by the Institutional Animal Care and Use Committee (IACUC, SNU-220311-1) of Seoul National University.

### Western blotting analysis

To analyze hADPN present in egg white (EW) from *OVAADPN* knock-in (KI) chickens, western blotting procedures were performed as previously described [29]. Briefly, for non-reducing conditions, samples were mixed with an equal volume of native sample buffer (BioRad, Hercules, CA, USA). For reducing conditions, samples were denatured by adding an equal volume of beta-mercaptoethanol (Sigma-Aldrich, St. Louis, MO, USA) in 2× Laemmli sample buffer (BioRad) and boiled at 95 °C for 5 min. In the western blotting analysis, EW samples were diluted to 500 times their volume and an equal volume of each sample was loaded. A total of 100 ng of commercial recombinant hADPN derived from human embryonic kidney (HEK) 293 cells (RD172023100; Biovendor R&D, Asheville, NC, USA), High-Five (Hi-5) cell-derived recombinant hADPN (450 − 24; PeproTech, Cranbury, NJ, USA), serum-derived commercial hADPN (CYT-024; ProSpec, East Brunswick, NJ, USA), or purified recombinant hADPN from *OVA ADPN* KI chicken EW was used for each analysis. Proteins were separated by sodium dodecyl sulfate polyacrylamide gel electrophoresis, transferred onto polyvinylidene fluoride membranes, and blocked with 5% skim milk (Becton Dickinson, East Rutherford, NJ, USA) for 1 h at room temperature. Subsequently, membranes were incubated with primary antibodies at 4 °C overnight, followed by appropriate horseradish peroxidase-conjugated secondary antibodies. Primary antibodies used in this study included anti-ADPN antibody (Abcam, Cambridge, UK) and anti-mouse IgG HRP-linked antibody (Santa Cruz Biotechnology, Dallas, TX, USA). Immunoreactive proteins were visualized using ECL Select Western Blotting Detection Reagent (Amersham, Buckinghamshire, UK), and signals were detected using a ChemiDoc XRS imaging system (BioRad).

### Quantification of ADPN protein

The quantities of total ADPN and HMW ADPN from *OVA ADPN* KI EW were measured using human total ADPN quantikine ELISA kit (DRP300; R&D systems, Minneapolis, MN, USA) and human HMW ADPN quantikine ELISA kit (DHWAD0; R&D systems) respectively. These assays were conducted following the manufacturer’s instructions and as described previously [29]. Briefly, these kits utilize the double-antibody sandwich method, where the optical density is proportional to the amount of anti-total ADPN or anti-HMW ADPN monoclonal antibody present. We determined the concentration of EW-derived hADPN by comparing with the optical density of the standard protein.

### Purification of hADPN from genome-edited hen EW

Purification of hADPN from *OVA ADPN* KI chicken EW was conducted according to previously established protocols [29]. Briefly, EW was combined with five times its volume of 40% ammonium sulfate (Thermo Fisher Scientific, Waltham, MA, USA) and stirred for 4 h at 4 °C. Subsequently, the mixture was centrifuged at 3000 g for 1 h at 4 °C, and the resulting pellet was resuspended in four volumes of 20 mM Tris-HCL and 50 mM NaCl (pH 8.0) to reach the original EW volume. The sample was filtered through a 0.2 μm bottle filter and loaded onto a 5 mL HiTrap Q column (Cytiva, Marlborough, MA, USA). The protein was eluted with 20 mM Tris-HCl and 1 M NaCl (pH 8.0), and further purified via size-exclusion chromatography using a Superdex 200 Increase 10/300 GL column (Cytiva) pre-equilibrated with 20 mM Tris-HCl and 50 mM NaCl (pH 8.0).

### Isolation of chicken adipose tissue and oviduct magnum

Wild-type 30-week-old White Leghorn and *OVA ADPN* KI hens were sacrificed for the isolation of chicken adipose tissue and oviduct magnum. Isolation of chicken adipose tissue was performed as described previously [30] with some adjustments. Briefly, the adipose tissues were separated from the subcutaneous fat tissues of the abdomen, washed with phosphate-buffered saline (PBS), and chopped into small pieces using a thin blade. Isolation of chicken oviduct magnum was conducted using a previously established method [31] with slight modifications. Magnum segments from the oviduct were trimmed and washed with PBS, the inner cell layer of the magnum was scraped using a thin blade, and collected tissues were dissolved in TRIzol reagent (Thermo Fisher Scientific) for subsequent RNA extraction.

### Cell culture and recombinant hADPN treatment

Human umbilical vein endothelial cells (HUVECs) were cultured in 12 well-plates (SPL Life Sciences, Pocheon, Korea) in a vascular cell basal medium (ATCC, Manassas, VA, USA). The culture medium comprised 0.2% bovine brain extract (BBE; ATCC), 5 ng/mL recombinant human epidermal growth factor (rhEGF; ATCC), 10 mM L-glutamine (ATCC), 0.75 Units/mL heparin sulfate (ATCC), 1 µg/mL hydrocortisone (ATCC), 50 µg/mL ascorbic acid (ATCC), 2% fetal bovine serum (ATCC), and 1× antibiotic-antimycotic agents (Thermo Fisher Scientific), and culturing was performed in a 5% CO_2_ incubator at 37 °C. Confluent cells were detached using 0.05% trypsin EDTA (Thermo Fisher Scientific) in Hank’s balanced salt solution (HBSS; Hyclone, Logan, UT, USA). For recombinant hADPN treatment, HUVECs under 10 passages were grown until 80−90% confluency, then treated with 10 µg/mL of EW-derived recombinant hADPN, HEK293 cell-derived commercial recombinant hADPN (Biovendor R&D), or Hi-5 cell-derived commercial recombinant hADPN (PeproTech) for 7 days. After treatment with recombinant hADPN, cells were subjected to stress induced by 100 µM H_2_O_2_ for 1 h.

### Oil Red O staining and quantification of Oil Red O

The treated HUVECs were fixed with 10% formalin for 1 h at room temperature. After washing with 60% isopropanol (1.09634.1011; Supelco, Bellefonte, PA, USA), cells were incubated with 0.2 μm filtered Oil Red O working solution (5694 − 4125; Daejung, Seoul, Korea) for up to 2 h with gentle rocking. Subsequently, cells with lipid droplets were imaged using an Eclipse Ti-U optical microscope (Nikon, Tokyo, Japan). To quantify accumulated lipid droplets, cells were washed with 60% isopropanol, Oil Red O stain was extracted with 100% isopropanol, and the absorbance was measured at 492 nm.

### Quantitative reverse transcription-polymerase chain reaction (RT-PCR)

Total RNA was extracted from HEK293 cells, chicken adipose tissue, chicken oviduct magnum, and recombinant hADPN-treated HUVECs by chopping and dissolving tissues in TRIzol reagent (Thermo Fisher Scientific). RNA was then reverse-transcribed using a SuperScript III Reverse Transcription Kit (Thermo Fisher Scientific), and the resulting complementary DNA (cDNA) was amplified using target gene-specific primer sets. Quantitative RT-PCR was performed with an initial denaturation at 95 °C for 5 min, followed by 40 cycles at 95 °C for 30 s, 60 °C for 30 s, and 72 °C for 1 min. Expression levels of the target genes were quantified using EvaGreen dye (Biotium, Hayward, CA, USA) and a StepOnePlus real-time PCR machine (Applied Biosystem, Waltham, MA, USA). Each test sample was analyzed in triplicate, and quantification of relative target gene expression was performed after normalization against the expression of chicken glyceraldehyde 3-phosphate dehydrogenase (*GAPDH*) as an endogenous control. Details about the primer sets used can be found in Supplementary Table 1. Human primer sets were used for HEK293 cell and HUVEC mRNA target gene amplification, and chicken primer sets were used for mRNA target gene amplification in chicken adipose tissue and the oviduct magnum.

### Statistical analyses

Statistical analysis was performed using Prism software (GraphPad, Boston, MA, USA). Significant differences between groups were determined by one-way analysis of variance (ANOVA) with Tukey’s multiple comparison tests for post-hoc analysis. A *p* < 0.05 was considered statistically significant.

## Results

### Sustainability of multimeric patterns in recombinant hADPN across subsequent generations of *ADPN* KI genome-edited chickens

**Fig. 1:**
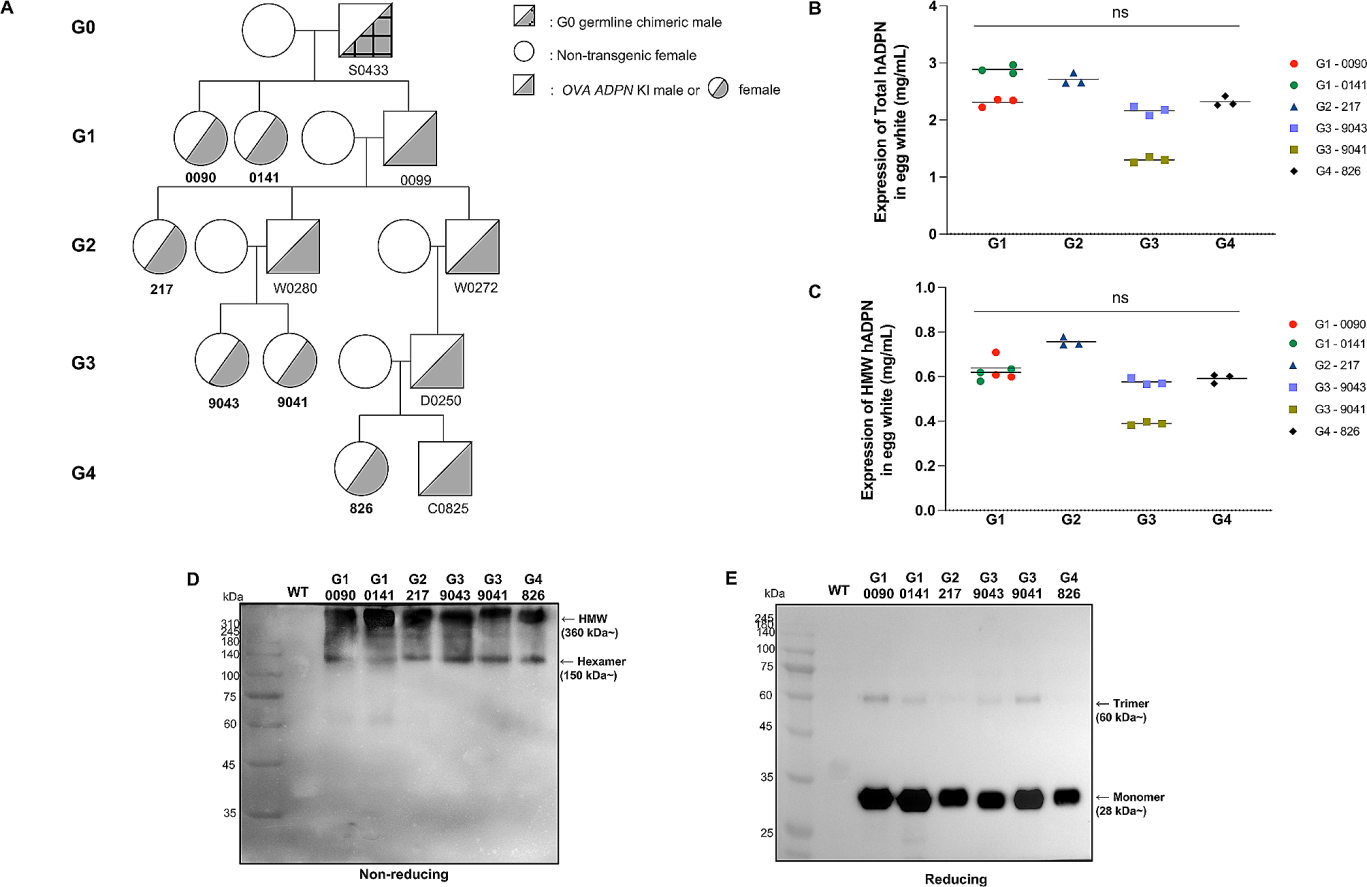
Sustainability of multimeric patterns in recombinant human adiponectin (hADPN) across subsequent generations of *ADPN* knock-in (KI) genome-edited chickens (**A**) Pedigree of ovalbumin (*OVA*) *ADPN KI* genome-edited chickens across generations. (**B**) Quantitative analysis of total ADPN protein abundance in genome-edited hens across generations. (**C**) Quantitative analysis of high molecular weight (HMW) ADPN protein abundance of genome-edited hens across generations. Statistical analysis was performed using one-way ANOVA (*n* = 3). Significant differences are indicated as “ns”, no significance. (**D**, **E**) Western blotting analysis of ADPN in *OVA ADPN* KI chicken egg white under non-reducing and reducing conditions. Arrows indicate the forms of ADPN, including HMW, hexamer (medium), trimer (low), and monomer

To ascertain the potential of *OVA ADPN* KI to serve as a stable and functional bioreactor system for continuous protein production, we utilized subsequent generations of heterozygous *OVA ADPN* KI genome-edited chickens from a previous study [29]. Protein content and oligomerization analyses were conducted on EW samples from various generations of genome-edited hens, including G1 (0090, 0141), G2 (217), G3 (9043, 9041), and G4 (826; Fig. 1A).

Quantitative expression levels of total ADPN across generations ranged from 1.30 mg/mL to 2.96 mg/mL, with an average of 2.28 mg/mL (Fig. 1B). HMW ADPN levels in genome-edited hens across generations ranged from 0.38 mg/mL to 0.78 mg/mL, with an average of 0.59 mg/mL (Fig. 1C). Notably, at G1, an average of 24% HMW ADPN was present in EW total ADPN. The HMW ADPN percentage was 28% at G2 and remained consistent at 28% at G3. At G4, the percentage was 25%. On average, ∼ 26% HMW ADPN was present in total ADPN in genome-edited hen EW across generations. All genome-edited hens in all generations exhibited similar levels of total and HMW ADPN expression without significant differences. There were variations among individual hens in G1 and G3 generations, consistent with observations in a previous study [29].

Subsequent oligomerization pattern analysis of hADPN protein in genome-edited hen EW across generations by western blotting revealed strong signals at 150 kDa (hexamer) and > 360 kDa (HMW) under non-reducing conditions (Fig. 1D). Under reducing conditions, a prominent monomeric signal at 28 kDa was detected, accompanied by a weak trimer signal around 60 kDa (Fig. 1E). These findings strongly indicate the predominance of multimeric forms of hADPN, particularly HMW ADPN, in *OVA ADPN* KI chickens EW, and importantly, this pattern remained consistent in subsequent generations.

### Comparative analysis of the multimeric composition of recombinant hADPN

**Fig. 2:**
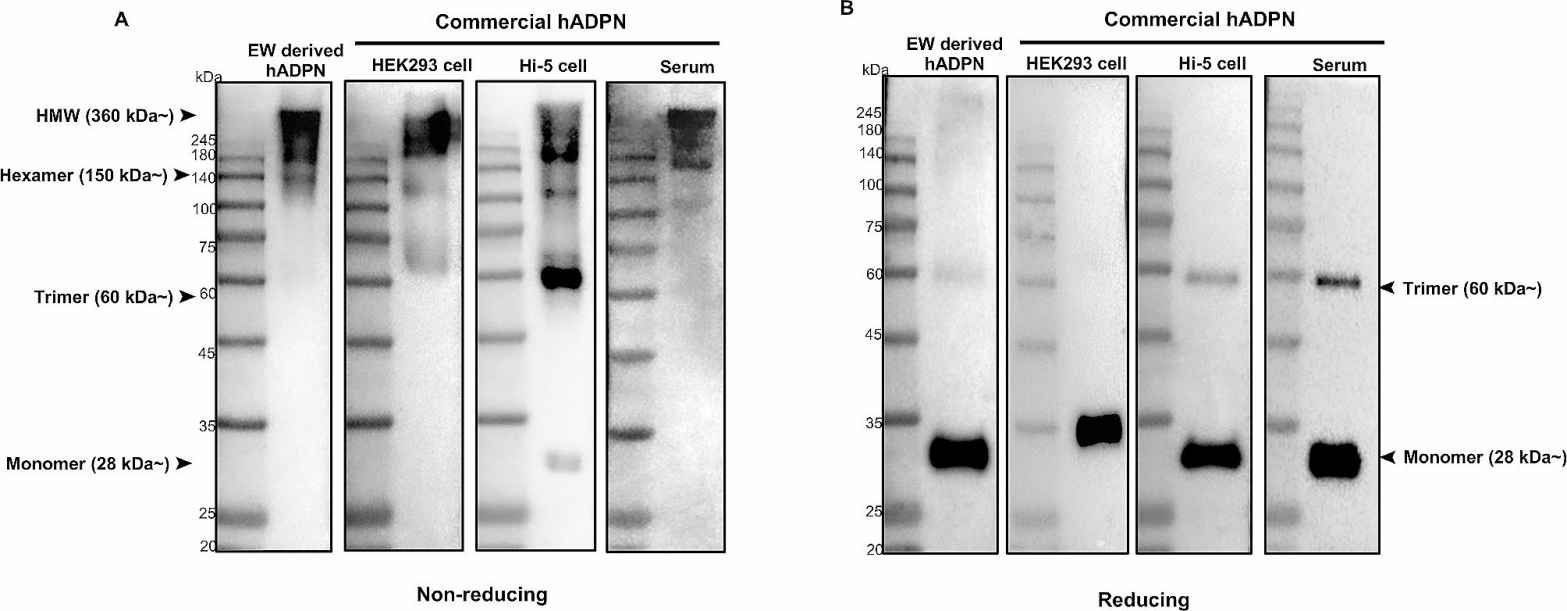
Comparative analysis of the multimeric composition of recombinant hADPN (**A**) Detection of multimeric recombinant hADPN derived from *OVA ADPN* KI chicken egg white (EW)-derived hADPN and other commercial recombinant hADPNs derived from human embryonic kidney (HEK) 293 cells with a C-terminal FLAG tag, High-Five (Hi-5) cells, and human serum (positive control) by western blotting analysis under non-reducing condition. (**B**) Detection of reduced EW-derived recombinant hADPN and other commercial recombinant hADPNs derived from HEK293 cells, Hi-5 cells, and human serum by western blotting analysis. Arrows indicate HMW, hexamer (medium), trimer (low), and monomer forms of hADPN.

To assess and compare the multimeric composition of hADPN generated by different recombinant systems with serum-derived hADPN, we purified hADPN from *OVA ADPN* KI EW and conducted a comparative analysis. We assessed genome-edited hen EW-derived recombinant hADPN and compared it with commercial recombinant hADPN derived from HEK293 and Hi-5 cells, as well as native serum-derived commercial hADPN as a positive control, using western blotting analysis. Under non-reducing conditions, EW-derived hADPN predominantly exhibited HMW forms and hexamers without trimers, closely resembling the multimeric pattern of serum-derived commercial hADPN. HEK293 cell-derived commercial hADPN displayed a mixture of hexamers and HMW forms with a trimer signal, while Hi-5 cell-derived commercial hADPN exhibited a strong trimer signal along with the presence of hexamers and a weak monomer signal (Fig. 2A).

Under reducing conditions, serum-derived commercial hADPN showed trimer and mostly monomer signals, similar to EW-derived hADPN and HEK293 cell-derived commercial hADPN, which displayed strong monomer signals. For HEK293 cell-derived commercial hADPN, the size was increased due to the C-terminal FLAG tag. Additionally, Hi-5 cell-derived commercial hADPN exhibited a strong monomer signal and a weak trimer signal (Fig. 2B). These results indicate that, under non-reducing conditions, *OVA ADPN* KI EW-derived recombinant hADPN closely resembled the multimeric pattern of native serum-derived hADPN, primarily composed of hexameric and HMW forms.

### Comparative analysis of endoplasmic reticulum (ER) chaperone gene transcription in the adipose tissue, oviduct magnum of wild-type (WT) and *ADPN* KI hens, and HEK293 cells

**Fig. 3:**
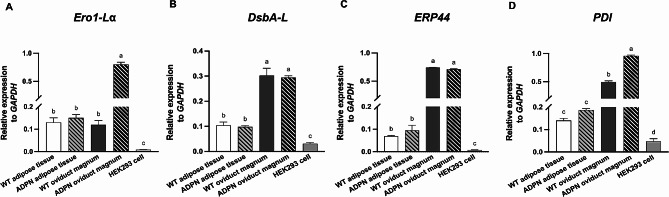
Comparative analysis of endoplasmic reticulum (ER) chaperone gene transcription in the adipose tissue, oviduct magnum of wild-type (WT) and *ADPN* KI hens, and HEK293 cells (**A**−**D**) mRNA expression levels of *Ero1-Lα*, *DsbA-L*, *ERP44*, and *PDI* in the adipose tissue and oviduct magnum of WT and *ADPN* KI hens compared with HEK293 cells. Samples from WT hens are denoted as WT adipose tissue and WT oviduct magnum, while samples from *ADPN* KI hens are labeled as ADPN adipose tissue and ADPN oviduct magnum. Statistical analysis was performed using one-way ANOVA (*n* = 3). Different superscript letters indicate significant differences between groups (*p* < 0.05)

Several ER chaperones are believed to play a role in the release of ADPN and the assembly of ADPN into its oligomeric forms [14, 32, 33]. To investigate the role of ER chaperones in the secretion and multimerization of hADPN into EW of *OVA ADPN* KI hens, we examined the mRNA expression levels of ER chaperone genes associated with the ADPN secretion and multimerization pathway across different samples, including chicken adipose tissue (the primary site of ADPN secretion) and the oviduct magnum (where EW proteins are synthesized) from both wild-type (WT) and *ADPN* KI hens, in compared with HEK293 cells (a commonly used mammalian recombinant system). Our analysis revealed notable differences in the expression of ER chaperones among the different samples. The mRNA expression level of *Ero1-Lα*, crucial for the secretion of HMW ADPN, showed no significant differences between the adipose tissues of WT and *ADPN* KI hens and the oviduct magnum of WT hens. However, it was significantly higher in the oviduct magnum of *ADPN* KI hens compared to the oviduct magnum of WT hens and the adipose tissues of both WT and *ADPN* KI hens (Fig. 3A).

 Similarly, the expression levels of *DsbA-L* and *ERP44*, which are involved in ADPN multimerization, did not significantly differ between WT and *ADPN* KI hens in the adipose tissue and oviduct magnum (Fig. 3B and C). Furthermore, the expression of *PDI*, essential for the intermolecular disulfide bond formation and adiponectin multimerization [15], was significantly higher in the oviduct magnum of *ADPN* KI hens compared to that of WT hens (Fig. 3D). However, both the adipose tissue and oviduct magnum of WT and *ADPN* KI hens exhibited significantly higher expression of all ER chaperone genes compared to HEK293 cells (Fig. 3). These findings suggest that the oviduct magnum of ADPN KI hens is highly active in ADPN secretion and multimerization processes, underscoring its potential as a reliable source of multimeric hADPN in a chicken bioreactor system.

### Comparative inhibitory effect of recombinant hADPN on lipid accumulation

**Fig. 4:**
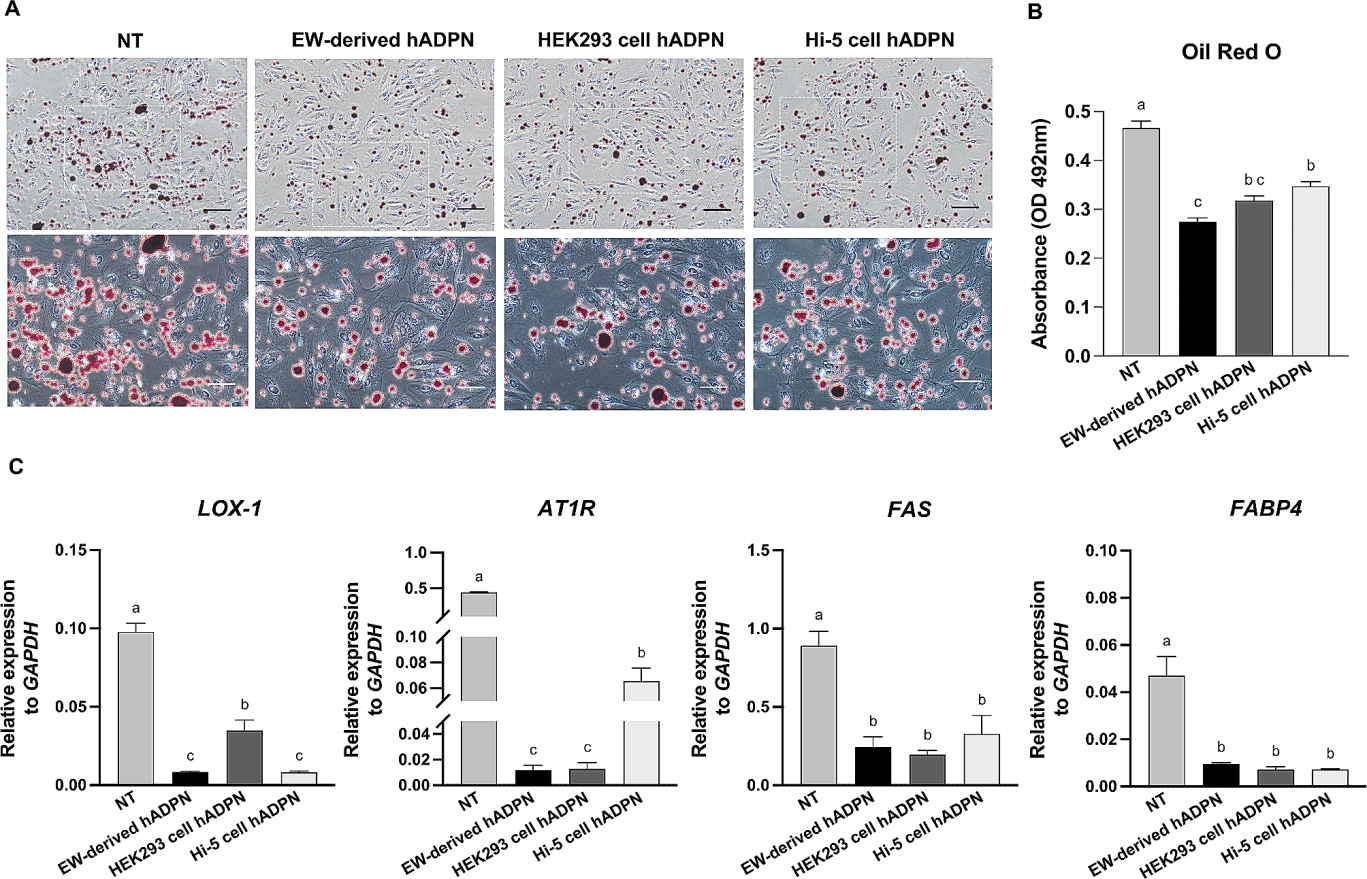
Comparative inhibitory effect of recombinant hADPNs on lipid accumulation (**A**) Representative images of Oil Red O-stained human umbilical vein endothelial cells (HUVECs) after treatment with 10 µg/mL EW-derived hADPN, HEK293 cell-derived commercial hADPN (HEK293 cell hADPN), or Hi-5 cell-derived commercial hADPN (Hi-5 cell hADPN) for 7 days, followed by 100 µM H_2_O_2_-induced stress for 1 h. NT, untreated. Scale bar = 100 μm (**B**) Quantification of extracted Oil Red O stain by spectrophotometry at 492 nm. (**C**) mRNA expression levels of *LOX-1*, *AT1R*, *FAS*, and *FABP4* in recombinant hADPN-treated HUVECs. Statistical analysis was performed using one-way ANOVA (*n* = 3). Different superscript letters indicate significant differences between groups (*p* < 0.05)

ADPN is known to play a protective role against atherosclerosis and lipid accumulation by inhibiting the uptake of LDL in endothelial cells [3, 34]. To assess the comparative functional effects on lipid accumulation in HUVECs, a commonly used in vitro model for studying the role of ADPN in endothelial cells, HUVECs were treated with either 10 µg/mL EW-derived hADPN, or other commercial recombinant hADPNs derived from HEK293 cells (HEK293 cell hADPN) or Hi-5 cells (Hi-5 cell hADPN) for 7 days. Following treatment, cells were subjected to short-term stress induced by H_2_O_2_ and subsequently stained with Oil Red O to visualize lipid droplet uptake. Untreated (NT) HUVECs displayed significantly higher accumulation of lipid droplets than the recombinant hADPN-treated groups (Fig. 4A).

 Quantification of accumulated lipid droplets revealed a significant reduction in cells treated with recombinant hADPNs compared to NT HUVECs. HUVECs treated with EW-derived hADPN exhibited a greater reduction in lipid accumulation than those treated with HEK293 cell hADPN or Hi-5 cell hADPN. The lipid droplet level in HUVECs treated with HEK293 cell hADPN was not significantly different from those in cells treated with Hi-5 cell hADPN or EW-derived hADPN. However, when compared to HUVECs treated with Hi-5 cell hADPN, those treated with EW-derived hADPN showed a significant decrease in lipid droplet content (Fig. 4B).

Endothelial uptake of oxidized LDL plays a critical role in atherosclerosis, and oxidized LDL accumulation upregulates *LOX-1* and *AT1R* [10, 35]. In addition, *FAS* and *FABP4* are associated with lipid synthesis, accumulation, and fat deposition [11, 36]. Our results indicated that mRNA expression levels of *LOX-1*, *AT1R*, *FAS*, and *FABP4* were significantly reduced in HUVEC groups treated with recombinant hADPN compared to NT HUVECs. Specifically, *LOX-1* expression was significantly lower in HUVECs treated with EW-derived hADPN or Hi-5 cell hADPN than HUVECs treated with HEK293 cell hADPN. Moreover, cells treated with EW-derived hADPN or HEK293 cell hADPN exhibited significantly lower *AT1R* expression compared to those treated with Hi-5 cell hADPN. However, there were no significant differences in the expression of *FAS* and *FABP4* among different recombinant hADPN-treated HUVEC groups (Fig. 4C). These findings suggest a comparative functional effect among recombinant hADPNs, with EW-derived hADPN demonstrating an enhanced inhibitory effect on lipid accumulation in HUVECs, indicating its potential therapeutic relevance.

## Discussion

The chicken egg has emerged as a highly efficient bioreactor for producing therapeutic proteins [37, 38], and recent advancements have led to the development of *OVA*-targeted genome-edited chickens capable of accumulating hADPN in egg white [29]. In this study, we conducted a comprehensive analysis of consistent production of stable multimeric hADPN across successive generations of *OVA ADPN* KI hens, highlighting the stability of multimeric patterns and the role of the oviduct magnum in producing HMW ADPN within a chicken bioreactor system. We also compared the functionality of EW-derived hADPN with other commercial hADPNs, particularly focusing on its ability to inhibit lipid accumulation.

Consistent expression levels of total and HMW ADPN were observed across generations of *OVA ADPN* KI hens, with an average of 26% HMW ADPN, despite individual variations (Fig. 1B and C). This stable expression aligns with previous studies utilizing the *OVA*-targeted KI system, underscoring its suitability for large-scale recombinant protein production [39]. Western blot analysis further highlighted the persistent presence of multimeric forms, particularly HMW ADPN, in *OVA ADPN* KI hen EW, indicating the potential for sustained multimeric hADPN production (Fig. 1D and E). EW-derived hADPN predominantly existed in HMW and multimeric form, similar to native serum-derived commercial hADPN, compared to commercial hADPN derived from HEK293 or Hi-5 cells (Fig. 2A). In contrast to traditional recombinant hADPN production methods using in *E. coli* or mammalian cells, our findings underscore the chicken bioreactor system as a promising alternative due to its abundance of HMW and MMW ADPN with active multimerization. We further investigated the molecular mechanisms underlying ADPN multimerization by comparing the expression levels of ER chaperone genes involved in this process. These ER chaperones play a crucial role in facilitating the assembly and secretion of HMW ADPN [13, 32]. Our results revealed higher expression levels of *DsbA-L*, *ERP44*, and *PDI* in the oviduct magnum than in the adipose tissue of both WT and *ADPN* KI hens (Fig. 3B and D). Notably, the expression levels of *Ero1-Lα* and *PDI* were upregulated in the oviduct magnum of *ADPN* KI hens compared to that of WT hens (Fig. 3A and D). The upregulation of these ER chaperone genes in the oviduct magnum may be attributed to the oviduct magnum-specific *OVA*-targeted KI of the *ADPN* gene. *Ero1-Lα*, *DsbA-L, ERP44*, and *PDI* were expressed significantly higher in the adipose tissue and oviduct magnum of both WT and *ADPN* KI hens compared to HEK293 cells (Fig. 3). These findings suggest that the oviduct magnum of *ADPN* KI hen is the optimal site for forming active multimeric hADPN.

In addition, we conducted functional investigations to evaluate the inhibitory effect of EW-derived hADPN on lipid accumulation in HUVECs. The results demonstrated that HUVECs treated with EW-derived hADPN, rich in hexamer and HMW multimers, displayed a significantly greater reduction in lipid accumulation than those treated with other commercial recombinant hADPNs (Fig. 4A and B). Notably, *LOX-1* plays a crucial role in internalizing oxidized LDL, which activates and expresses *LOX-1* upon uptake of oxidized LDL [9]. It is reported that oxidized LDL upregulates *AT1R* mRNA via NADPH oxidase-mitogen-activated protein kinase-nuclear factor-kappaB pathway, and Ang II further upregulates *LOX-1* expression in a positive feedback loop [10]. Furthermore, key genes in lipid synthesis and cellular lipid transport, such as *FAS* and *FABP4* [9, 12, 40], underscore the importance of ADPN in protecting against oxidized LDL accumulation and suppressing genes related to lipid synthesis [3, 41, 42]. Given the abundance of hexamer and HMW multimers in EW-derived hADPN, our findings highlight its significantly lower lipid droplet content compared to NT HUVECs and those treated with Hi-5 cell-derived commercial hADPN. Additionally, EW-derived hADPN-treated cells exhibited significantly lower expression of *AT1R* and *LOX-1* than NT cells and those treated with other commercial recombinant hADPNs (Fig. 4C). These results suggest that the inhibitory effect of EW-derived hADPN on lipid accumulation in endothelial cells, indicating its potential therapeutic relevance.

In summary, our study provides insights into the amount and multimerization pattern of hADPN across several generations of *OVA ADPN* KI chickens. We also elucidate multimerization-related gene expression in the oviduct magnum of *ADPN* KI genome-edited chickens and the functionality of EW-derived hADPN in HUVECs.

## Conclusion

The results provide insight into the stability of multimeric patterns, the environmentally favorable tissue of the chicken oviduct magnum as a bioreactor for multimeric hADPN production, and the enhanced inhibitory function of EW-derived hADPN against lipid accumulation due to abundant hexameric and HMW forms compared with other commercial recombinant hADPNs. This information is invaluable for the development of biotechnological applications and the potential therapeutic use of multimeric hADPN. Future research should explore the practical application of these findings for producing therapeutic proteins in a more cost-effective and efficient manner.

### Electronic supplementary material

Below is the link to the electronic supplementary material.

Supplementary Material 1

## Data Availability

All data generated or analyzed during this study are included in this published article and its supplementary information files.
