# Supplementary material for: Sustainable production of multimeric and functional recombinant human adiponectin using genome-edited chickens
Source: J Biol Eng. 2024 May 7;18:32. doi: 10.1186/s13036-024-00427-2 (PMC11077872; doi:10.1186/s13036-024-00427-2)
Supplement: Supplementary file 1 — Supplementary Material 1 [file 13036_2024_427_MOESM1_ESM.docx]

**Supplementary table S1. Oligonucleotide sequences used in this study.**

| ID | Sequence (5’ to 3’) | Usage |
| --- | --- | --- |
| Chicken *GAPDH* F | **TGG TGG CCA TCA ATG ATC CC** | **quantitative RT-PCR** |
| Chicken *GAPDH* R | **CCG TTC TCA GCC TTG ACA GT** | **quantitative RT-PCR** |
| Chicken *Ero1-Lα* F | **CCA GGC ATC AGG TCC TTT GT** | **quantitative RT-PCR** |
| Chicken *Ero1-Lα* R | **AGC CGT GCT GAA GAA TGT GA** | **quantitative RT-PCR** |
| Chicken *DsbA-L* F | **ATG CGT TTT ATC ACA GCC GC** | **quantitative RT-PCR** |
| Chicken *DsbA-L* R | **ACG ATT CTT CAC TGC AGG GG** | **quantitative RT-PCR** |
| Chicken *ERP44* F | **AAT ACA GGG GCC AGA GGT CT** | **quantitative RT-PCR** |
| Chicken *ERP44* R | **CCC CAA AGG CAG ACA GGA AT** | **quantitative RT-PCR** |
| Chicken *PDI* F | **CTG GCA GGG AAG CAG ATG AT** | **quantitative RT-PCR** |
| Chicken *PDI* R | **ACT TGG AGA AGA CGT CAG CG** | **quantitative RT-PCR** |
| Human *GAPDH* F | **GGT CAT CCC TGA GCT GAA CG** | **quantitative RT-PCR** |
| Human *GAPDH* R | **AAA GTG GTC GTT GAG GGC AA** | **quantitative RT-PCR** |
| Human *Ero1-Lα* F | **GAA GGC TGT TCT TCA GTG GAC C** | **quantitative RT-PCR** |
| Human *Ero1-Lα* R | **CCC TTG TAA CCA GTG TAG CGC T** | **quantitative RT-PCR** |
| Human *DsbA-L* F | **AAG CTC CTG AGA CAC CAT CTC C** | **quantitative RT-PCR** |
| Human *DsbA-L* R | **CTC TGG ATG CTC CAA GTT CAC G** | **quantitative RT-PCR** |
| Human *ERP44* F | **TGC TGA CTG GTG TCG TTT CA** | **quantitative RT-PCR** |
| Human *ERP44* R | **TTT CAC TGA TCG CTG CC CC** | **quantitative RT-PCR** |
| Human *PDI* F | **TCA CCA AGG AGA ACC TGC TGG A** | **quantitative RT-PCR** |
| Human *PDI* R | **GGC AAG AAC AGC AGG ATG TGA G** | **quantitative RT-PCR** |
| Human *LOX-1* F | **GGA CCA GCC TGA TGA GAA GT** | **quantitative RT-PCR** |
| Human *LOX-1* R | **GGC TGA GAT CTG TCC CTC CA** | **quantitative RT-PCR** |
| Human *AT1R* F | **CAG CGT TTT CAA CCT GTA CG** | **quantitative RT-PCR** |
| Human *AT1R* R | **GCA GGT GAC TTT GGC TAC AAG C** | **quantitative RT-PCR** |
| Human *FAS* F | **TTC TAC GGC TCC ACG CTC TTC C** | **quantitative RT-PCR** |
| Human *FAS* R | **GAA GAG TCT TCG TCA GCC AGG A** | **quantitative RT-PCR** |
| Human *FABP4* F | **GCT TTG CCA CCA GGA AAG TG** | **quantitative RT-PCR** |
| Human *FABP4* R | **CAC ATG TAC CAG GAC ACC CC** | **quantitative RT-PCR** |
